# Estimating the cost of not setting the Health Promotion Levy at 20% in South Africa: an extended cost-effectiveness analysis

**DOI:** 10.1136/bmjph-2025-004415

**Published:** 2026-07-09

**Authors:** Chengetai Dare, Evelyn Thsehla, Susan Goldstein, Micheal Kofi Boachie

**Affiliations:** 1 SAMRC/Wits Centre for Health Economics and Decision Science – PRICELESS SA (A Division of Wits Health Consortium), School of Public Health, Faculty of Health Sciences, University of the Witwatersrand Johannesburg, Johannesburg, South Africa

**Keywords:** Diabetes Mellitus, Public Health, Obesity, Program Evaluation

## Abstract

**Introduction:**

Taxes on sugar-sweetened beverages are recommended by the WHO to reduce consumption and improve population health. In South Africa, the Health Promotion Levy (HPL) was introduced at an effective rate of approximately 11% but has declined to about 8% in real terms due to inflation, remaining below the WHO-recommended 20% threshold. This study evaluates the health and fiscal implications of maintaining the HPL below this level, focusing on type 2 diabetes outcomes.

**Methods:**

We conducted an extended cost-effectiveness analysis using a proportional multistate life table model to simulate the impact of increasing the HPL from its current level to an effective 20% over a 20-year period (2022–2042). Data were drawn from the National Income Dynamics Survey, All Media and Products Survey and the Global Burden of Disease Study. Outcomes included diabetes incidence, prevalence, mortality, healthcare costs and tax revenue across income quintiles.

**Results:**

Increasing the HPL to 20% could avert 619 000 incident cases of type 2 diabetes, 285 000 prevalent cases and 26 000 deaths over 20 years, while saving ZAR23.9 billion in healthcare costs compared with maintaining the current rate. Sensitivity analyses (80%–120% passthrough) yielded 514 000–659 000 cases averted. Maintaining the current HPL resulted in ZAR15.1 billion in forgone revenue. Health gains were greatest in absolute terms among females and higher-income groups, but benefits were observed across all quintiles.

**Conclusions:**

Maintaining the HPL below the WHO-recommended level represents a missed opportunity to reduce the burden of type 2 diabetes and generate additional healthcare savings and revenue in South Africa. Increasing the HPL to 20% could deliver substantial health and fiscal benefits and should be considered as part of broader strategies to address the growing burden of non-communicable diseases.

**WHAT IS ALREADY KNOWN ON THIS TOPIC**

- Taxes on sugar-sweetened beverages reduce consumption and improve health, but South Africa’s Health Promotion Levy remains below the WHO’s recommended 20% threshold.

**WHAT THIS STUDY ADDS**

- This study quantifies the health and fiscal consequences of maintaining the Health Promotion Levy (HPL) below the recommended threshold, estimating substantial missed opportunities in terms of type 2 diabetes cases averted, healthcare cost savings and tax revenue, while showing that benefits accrue across all income groups but are larger in absolute terms among higher-income populations.

**HOW THIS STUDY MIGHT AFFECT RESEARCH, PRACTICE OR POLICY**

- These findings support increasing the HPL to align with global recommendations and highlight the importance of considering both health and fiscal outcomes, as well as distributional effects, in the design and evaluation of health taxes.

## Introduction

 The global prevalence of overweight and obesity has increased substantially over the past four decades. In sub-Saharan Africa, the prevalence rates increased from 9% to 23% for men and from 17% to 39% for women between 1990 and 2022.^1^ In South Africa, the prevalence is among the highest in the region. In 2016, 31% of adult males, 67% of adult females and 13% of children under 5 years old were either overweight or obese.^2 3^ It is estimated that, in the absence of any intervention, 50% of South Africans will be obese by 2030.^4^ Overweight and obesity are major risk factors for type 2 diabetes mellitus and other non-communicable diseases (NCDs), such as hypertension, stroke, cardiovascular diseases and several cancers.^5^

In South Africa, the age-standardised prevalence of diabetes increased from 4.7% to 9.7% among men and 7.7 to 12.6% among women between 1980 and 2014.^6^ In 2021, approximately 4.2 million people were living with diabetes. Diabetes ranks second among the top ten underlying causes of death, with an average of 20 000 deaths annually between 2010 and 2018.^6^ In addition to its health burden, diabetes imposes substantial economic costs, estimated to range from ZAR2.7 billion in 2018 to ZAR29 billion in 2020.^7^,^9^ Although many statistics on diabetes do not differentiate between diabetes types, type 2 diabetes is estimated to account for approximately 90% of all diabetes cases.^7 10^ Importantly, type 2 diabetes can be prevented or delayed, including through reduction in sugar intake.^4 11 12^

One of the primary contributors to excess sugar intake is the consumption of sugar-sweetened beverages (SSBs), which are a major source of free sugars in the diet. SSBs are defined as non-alcoholic beverages containing added caloric sweeteners, including carbonated drinks, fruit drinks, energy drinks and sweetened teas.^13^ Evidence from South Africa indicates high levels of sugar consumption, with added sugar intake increasing substantially between 2005 and 2010 across both rural and urban populations.^14 15^ Liquid sugar is of particular concern due to its limited satiety effects and strong association with weight gain and type 2 diabetes risk.

In response to rising obesity and its comorbidities, the South African government announced the introduction of a tax on SSBs in 2016. The policy, formally known as the Health Promotion Levy (HPL), was initially proposed at ZAR0.028 per gram of sugar (approximately ZAR16.7=US$1 as of May 2026), equivalent to approximately 20% of the retail price of the most popular SSB in line with WHO recommendations.^16^,^18^ However, following extensive consultation with industry stakeholders, the tax was implemented at a lower rate. Introduced on 1 April 2018, the HPL was set at ZAR0.021 per gram of sugar above a threshold of 4 g per 100 mL of SSBs, corresponding to an effective tax burden of approximately 11%. In 2019, the levy was increased slightly to ZAR0.0221, but it has not been adjusted since then. As a result, due to inflation, the effective tax burden has declined to approximately 8% in real terms as of June 2025.

Evidence suggests that the effectiveness of excise taxes on unhealthy products depends on their magnitude and their ability to increase retail prices sufficiently to influence consumption behaviour.^18 19^ Maintaining the HPL below the WHO-recommended 20% threshold may therefore limit its potential to reduce SSB consumption and improve health outcomes.

This study aims to estimate the health and fiscal costs associated with maintaining South Africa’s HPL below the WHO-recommended 20% threshold, using type 2 diabetes as a case study. Specifically, we assess the potential health outcomes, healthcare cost implications and forgone tax revenues across income groups using an extended cost-effectiveness analysis (ECEA).

## Methods

This study employed ECEA methods to estimate the additional health costs associated with morbidity, mortality and prevalence of diabetes, and potential revenue lost, as a result of applying the HPL below the 20% effective tax burden. The model was adapted from Boachie *et al*^15^ and Saxena *et al*^20^ on related studies. Similar models have been widely used in evaluating health and economic impacts of health taxes.^21^,^23^

### Analytical framework

**Figure 1:**
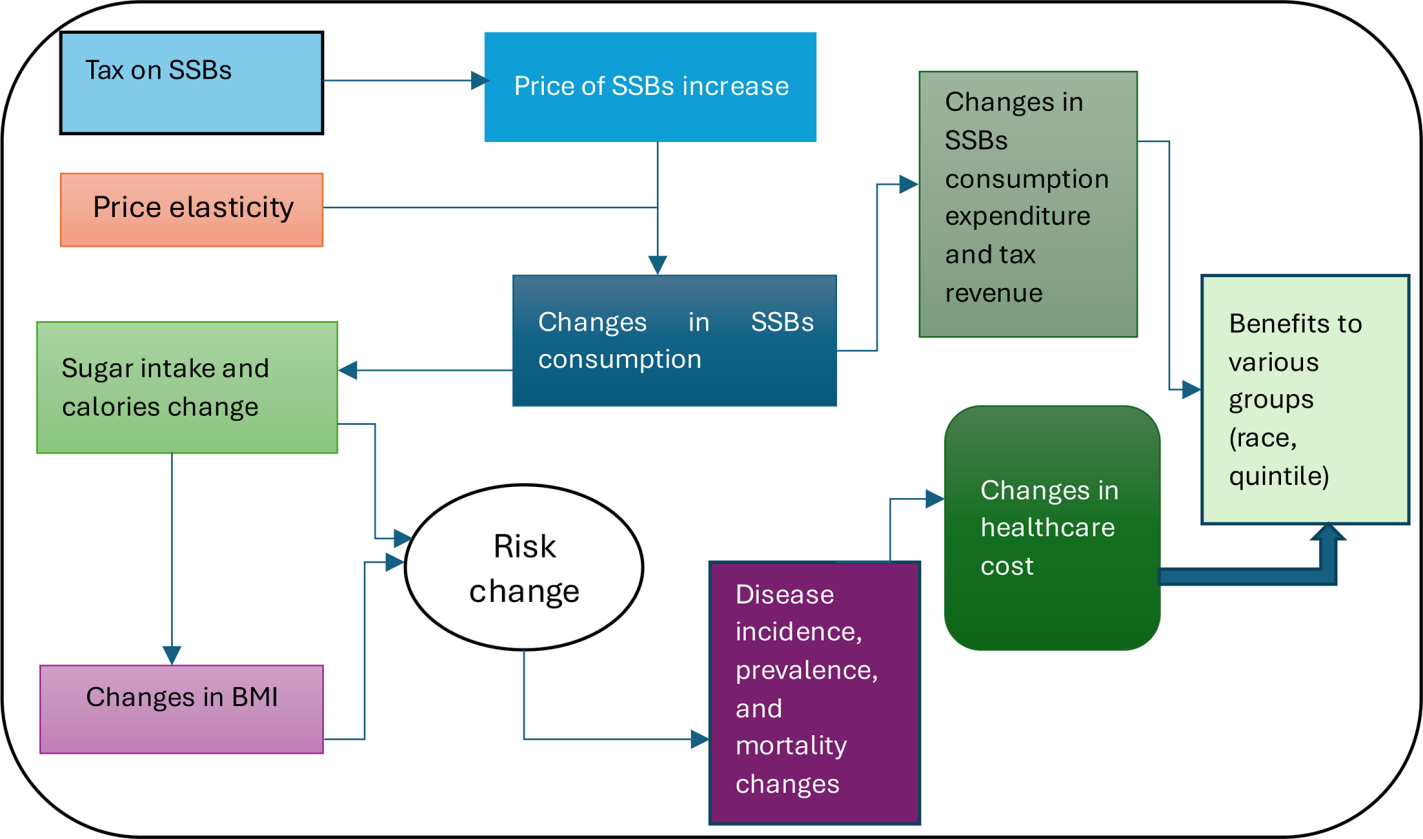
Conceptual framework of the link between sugar-sweetened beverage taxation and outcomes Adapted from Boachie *et al.*^15^ BMI, body mass index; SSBs, sugar-sweetened beverages.

Conventional economic theory suggests that higher taxes will lead to higher retail prices and consequently reduce SSB consumption, resulting in improved health and better financial outcomes for the population. Thus, the analytical framework of the tax-health nexus was based on the link between SSB consumption and weight gain or body mass index (BMI), with the magnitude of this interaction being mediated by the price elasticity of consumers’ demand. Figure 1 provides the conceptual framework for our analysis.

### Data sources

This study used data compiled from several sources. Total population (by age and sex) was sourced from the Stats SA mid-year population estimates for 2022.^24^ Data on height, weight and BMI, income distribution and the subsidised patients were sourced from the National Income Dynamics Survey Wave 5.^25^ SSB consumption data were estimated from All Media and Products Survey,^26^ assuming each can, bottle, glass or carton contained 330 mL liquid.^20^ Consumption was estimated by age, sex and income quintile. Price elasticity estimates were obtained from Saxena *et al.*^20^ Baseline data on age-specific and sex-specific incidence, prevalence and case-fatality rates of the disease were derived from the DisMod II software package using data from the Global Burden of Disease Study.^27^ Mortality probabilities and other lifetable variables were sourced from WHO.^28^ Table 1 depicts the main baseline characteristics and the inputs.

**Table 1:** Summary of main model input parameters

	Quintile 1	Quintile 2	Quintile 3	Quintile 4	Quintile 5	Source
Price elasticity	−1.26	−1.20	−1.20	−1.14	−0.98	20
Per capita income (monthly), ZAR	9119	11 771	17 716	31 612	114 311	24
Healthcare utilisation, %	45	50	65	70	80	20
Mean BMI (females)	21.86	22.17	22.59	23.19	24.84	25
Mean BMI (males)	26.18	26.90	27.27	27.77	28.08	25
Mean consumption, litres per week (females)	2.61	2.82	2.94	3.10	3.02	26
Mean consumption, litres per week (males)	2.70	3.31	3.53	3.77	3.84	26
Prevalence of diabetes	10.12%	12.15%	12.23%	13.25%	15.26%	27
Prevalence of SSB consumption	40%	44%	51%	60%	64%	48
Current tax rate for SSBs (modelled tax rate), ZAR/g	0.0221 (0.083)					49 50
Sugar content per litre	106					51
Expected tax-free sugar content/litre	40					49 51
Retail price per litre, ZAR	20.89					52

Notes: All prices and incomes are inflation-adjusted to 2022.

BMI, body mass index; SSBs, sugar-sweetened beverages.

### Estimation methods

Following previous studies,^15 20 29^ the rand value of the HPL was converted to a percentage based on the average retail price of a litre of SSBs in South Africa. The ZAR0.0221 was (in June 2025) equivalent to 8.3% of the average retail price of a litre of SSB, while the 20% tax burden translates into ZAR0.0545. The percentage change in the retail price was multiplied by quintile-specific price elasticities to obtain the percentage change in quantity of SSB consumed.^15^

Previously published mathematical obesity models^1529^,^31^ were adapted to estimate the effect of reduced consumption of SSB on type 2 diabetes incidence over a 20-year period (2022–2042). These models use proportional multistate lifetable in nature which assumes disease independence in a Markov modelling framework.^15 32^ The estimation process follows two broad steps. First, change in consumption is converted into change in energy intake and translated into impact on body weight. In line with existing literature, it was assumed that SSB has an energy density of 1340 kilojoules (kJ) per litre.^15 31^ Changes in consumption and energy intake were then converted into change in body weight using an energy balance equation that a daily energy change of 94 kJ was associated with a change of 1 kg in body weight for adults.^15 33^ This assumed that physical activity levels remain unchanged. The change in body weight and average height of individuals in each age-quintile category was used to obtain the change in age-quintile specific BMI. The trend of BMI was modelled as a lognormal distribution over 20 years.^15^

The second step involved translating changes in BMI into incidence of type 2 diabetes mellitus using the potential impact fraction (PIF), a measure of how much the risk of disease changes when the exposure to a risk factor changes. The age-and sex-specific PIF were estimated using data on the relative risk of type 2 diabetes mellitus due to a unit increase in BMI and the change in BMI, using the EpigearXL add-in for Microsoft Excel, version 14.0. The baseline incidence rate was scaled by the PIF to obtain the incidence and mortality rate that resulted from raising the HPL to 20% of the retail price. The changes in the incidence and mortality rates due to the HPL adjustment then formed the inputs into the cohort lifetables. The population was simulated from age two to 100 years of lifetime or death, whichever comes first,^20 29 30^ to estimate the cohort-specific changes in type 2 diabetes incidence, premature deaths and healthcare costs over a 20-year period using Erstaz add-in to Microsoft Excel V.14.0. The study modelled the 2022 mid-year population (25 years and above) for South Africa.

The potential tax revenue loss to the government as a result of setting the effective tax rate of the HPL below the 20% threshold was then estimated. The revenue loss was estimated for the period 2018/2019–2024/2025, by income quintile. Using the HPL revenue data from National Treasury, the amount of sugar (in grams) levied was calculated, and multiplied the same by the tax amount at the 20% tax rate to obtain the potential revenue. The revenue loss is the difference between the potential revenue (at 20% effective tax rate) and the actual revenue collections.

As suggested by economic theory, higher taxes lead to higher retail prices and consequently reduce SSB consumption, resulting in improved health and better financial outcomes for the population. Consumers change their purchasing behaviour in response to retail price changes, not in response to tax changes.^34^ The effectiveness of the HPL to regulate SSB intake ultimately depends on how taxes are passed through to prices (ie, whether taxes sufficiently raise prices).

Producers may respond to tax policies differently, depending on their market power and profit maximisation incentives. They may raise the price by less than the tax increase, which is known as tax under-shifting. Producers may raise the price by the exact amount of the tax increase (exactly shifting or fully passed-through). Alternatively, taxes may be over-shifted (ie, prices increase by more than the tax increase). The degree of passthrough thus determines the effectiveness of the excise tax in reducing SSB consumption (apart from the hypothetical cases in which demand or supply is perfectly price-inelastic).^34^ For example, a fully passed-through tax increase is more effective in reducing consumption than an undershifted tax increase, while an overshifted tax increase would be more effective than an equally-shifted tax. Based on Stacey *et al*,^35^ in this study tax passthrough was pegged at 100% (exact shifting).

### Sensitivity analysis

Due to uncertainties surrounding the degree of the tax passthrough, univariate sensitivity analyses were performed to understand the effects of the variation in tax passthroughs and their subsequent impacts on the study outcomes. The tax passthroughs were varied from 100% to 80% and 120%, across all income quintiles in line with Boachie *et al*^15^ on a related study.

### Patient and public involvement

None.

## Results

For the period 2018/2019–2023/2024, a total of ZAR30.3 billion would have been collected had the HPL been levied at an effective tax rate of 20%. The highest amount of potential revenue (ZAR8 billion) is recorded in 2018/2019, while the lowest (ZAR2.8 billion) is registered in 2023/2024. However, the total actual collections amounted to ZAR15.1 billion, resulting in a potential revenue loss of ZAR15.1 billion. Accordingly, the highest and lowest amounts of revenue forgone are registered in 2018/2019 and 2023/2024. By income group, the lowest amount of revenue forgone (ZAR4.2 billion) is registered in quintile 1, while the largest amounts (ZAR5.4 billion) are registered in quintiles 4 and 5. The amounts of potential revenue (at 20% effective tax rate), actual tax collections and the forgone tax revenue, by quintile and fiscal year, are shown in table 2.

**Table 2:** HPL revenue forgone, 2018/2019–2023/2024

	Potential tax revenue (ZAR million)	Actual tax revenue (ZAR million)	Revenue forgone (ZAR million)	Revenue forgone, by quintile (ZAR million)				
				Quintile 1	Quintile 2	Quintile 3	Quintile 4	Quintile 5
2018/2019	8025.0	3248.2	4776.8	800.2	927.0	976.8	1037.2	1035.7
2019/2020	6498.7	2512.9	3985.8	667.7	773.5	815.0	865.4	864.2
2020/2021	5262.7	2113.6	3149.1	527.5	611.1	643.9	683.8	682.8
2021/2022	4261.7	2259.8	2001.9	335.3	388.5	409.4	434.7	434.0
2022/2023	3451.2	2429.9	1021.3	171.1	198.2	208.8	221.7	221.4
2023/2024	2794.8	2583.5	211.3	35.4	41.0	43.2	45.9	45.8
Total	30 294.0	15 147.9	15 146.1	2537.1	2939.2	3097.2	3288.7	3283.9

HPL, Health Promotion Levy.

Table 3 shows the health and financial effects associated with setting the HPL at 20% and 11%, and the associated cost of inaction (ie, the cost not setting the HPL at 20%) over a 20-year period. The negative values signify averted incident cases, prevalence, deaths and health costs. The first segment of the table shows the estimates disaggregated by gender while the second segment shows the estimates in aggregated form. The results show that increasing the HPL from 11% to 20% effective tax rate significantly reduces incident cases of type 2 diabetes, prevalence, mortality and health costs across all quintiles. The greatest impact is registered in quintile 5, followed by quintiles 1, 4, 3 and 2. For instance, in quintile 5, a 11% effective rate reduces the incident and prevalent cases by 157 000 and 77 000, respectively. Setting the effective tax rate at 20% reduces the number of incident and prevalent cases of type 2 diabetes by 324 000 and 153 000, respectively. Thus, raising the effective tax rate from 11% to 20% averts an additional 167 000 incident and 76 000 prevalent cases. Also, the 20% effective tax burden further averts 7 000 mortality cases while reducing health costs by ZAR7 billion over a period of 20 years. Overall, a 20% increase in the effective tax rate averts additional incident cases, prevalence, mortality and health costs by 619 000, 285 000, 26 000 and ZAR23.9 billion, respectively, over 20 years.

**Table 3:** Estimates of the potential effect of not setting the HPL at 20% effective tax rate at 100% tax passthrough

By gender													
Quintile	Gender	At 20% effective tax rate				At 11% effective tax rate				Cost of inaction			
		Incident cases	Prevalence	Deaths	Health costs (ZAR)	Incident cases	Prevalence	Deaths	Health costs (ZAR)	Incident cases	Prevalence	Deaths	Health costs (ZAR)
1	Male	−108 893	−42 636	−4660	−3 983 138 820	−55 658	−22 521	−2395	−2 100 095 353	−53 235	−20 116	−2265	−1 883 043 467
	Female	−235 762	−126 999	−9461	−9 930 811 363	−139 977	−77 404	−5469	−6 089 934 195	−95 784	−49 596	−3992	−3 840 877 168
2	Male	−66 262	−26 064	−2855	−2 371 241 957	−39 882	−16 170	−1751	−1 471 223 452	−26 380	−9893	−1104	−900 018 505
	Female	−150 697	−77 528	−6775	−6 406 035 736	−92 698	−49 482	−4212	−4 088 631 559	−57 999	−28 046	−2563	−2 317 404 178
3	Male	−71 906	−28 598	−3080	−2 581 617 407	−39 001	−16 049	−1711	−1 455 698 022	−32 904	−12 549	−1369	−1 125 919 386
	Female	−146 343	−76 751	−6257	−6 151 389 104	−81 527	−44 612	−3516	−3 581 599 625	−64 817	−32 139	−2740	−2 569 789 479
4	Male	−81 840	−32 548	−3484	−2 981 356 252	−38 643	−15 931	−1705	−1 467 476 389	−43 196	−16 617	−1779	−1 513 879 863
	Female	−156 301	−83 879	−6379	−6 655 008 336	−78 108	−43 796	−3253	−3 497 663 697	−78 193	−40 084	−3126	−3 157 344 639
5	Male	−123 390	−46 899	−5482	−4 613 002 033	−60 458	−23 856	−2757	−2 350 759 365	−62 932	−23 043	−2725	−2 262 242 668
	Female	−200 568	−105 961	−8510	−8 772 662 743	−96 967	−53 222	−4257	−4 442 984 908	−103 601	−52 739	−4252	−4 329 677 835
Overall		−1 341 962	−647 865	−56 943	−54 446 263 751	−722 919	−363 044	−31 027	−30 546 066 563	−619 043	−284 821	−25 916	−23 900 197 188

Total (sum of males and females)												
Quintile	At 20% effective tax rate				At 11% effective tax rate				Cost of inaction			
	Incident cases	Prevalence	Deaths	Health costs (ZAR)	Incident cases	Prevalence	Deaths	Health costs (ZAR)	Incident cases	Prevalence	Deaths	Health costs (ZAR)
1	−344 655	−169 636	−14 122	−13 913 950 183	−195 635	−99 925	−7864	−8 190 029 547	−149 020	−69 711	−6258	−5 723 920 635
2	−216 960	−103 592	−9630	−8 777 277 693	−132 580	−65 652	−5963	−5 559 855 011	−84 380	−37 940	−3667	−3 217 422 683
3	−218 249	−105 350	−9336	−8 733 006 511	−120 528	−60 662	−5227	−5 037 297 646	−97 721	−44 688	−4109	−3 695 708 865
4	−238 141	−116 427	−9863	−9 636 364 588	−116 751	−59 726	−4958	−4 965 140 085	−121 390	−56 701	−4905	−4 671 224 502
5	−323 958	−152 860	−13 992	−13 385 664 776	−157 425	−77 079	−7015	−6 793 744 273	−166 533	−75 782	−6977	−6 591 920 503
Overall	−1 341 962	−647 865	−56 943	−54 446 263 751	−722 919	−363 044	−31 027	−30 546 066 563	−619 043	−284 821	−25 916	−23 900 197 188

HPL, Health Promotion Levy.

For sensitivity analysis, the tax passthrough was varied from 100% to 80% and 120%. The results are depicted in online supplemental tables S1 and S2. Under the 80% tax passthrough, raising the HPL to 20% averts 514 000 incident cases of type 2 diabetes, 240 000 prevalent cases, 21 000 deaths and ZAR20.1 billion in health costs. Relatedly, a 20% increase in HPL when the tax passthrough is 120% averts additional 659 000 incident cases of type 2 diabetes, 299 000 prevalent cases, 28 000 deaths and ZAR25.3 billion in health costs.

## Discussion

This study provides an analysis of the health and fiscal implications of maintaining South Africa’s HPL on SSBs at an effective tax rate of 11%, significantly below the WHO-recommended minimum threshold of 20%. By employing an ECEA, it is estimated that failing to increase the HPL to 20% of retail price results in several missed opportunities: 619 000 additional incident cases of type 2 diabetes, 285 000 prevalent cases, 26 000 premature deaths and ZAR23.9 billion in healthcare costs over a 20-year period (2022–2042). Additionally, the government has forgone ZAR15.1 billion in potential tax revenue between 2018/2019 and 2023/2024. These findings underscore the critical role of evidence-based fiscal policies in addressing South Africa’s escalating NCD epidemic, particularly type 2 diabetes, which ranks as the second leading cause of mortality in the country.^6^

The health benefits of raising the HPL to 20% include the potential to avert 619 000 incident cases of type 2 diabetes over two decades. Notably, 60% of these health benefits accrue to females, reflecting the higher prevalence of obesity and type 2 diabetes among women in South Africa, where 67% of adult females were overweight or obese in 2016 compared with 31% of males.^2 3^ This gender disparity aligns with global trends, where women often face higher obesity rates due to socioeconomic factors, including limited access to healthy foods and opportunities for physical activity.^36^

An important consideration is how these benefits are distributed across income groups. The greatest absolute health benefits are observed in Quintile 5 (highest income group), driven by higher SSB consumption and lower price elasticity (−0.98), indicating greater responsiveness to price changes.^20^ However, meaningful health gains are also observed in quintile 1 (lowest income group), including reductions in diabetes incidence and associated healthcare costs. These findings suggest a distributionally mixed effect, rather than a strictly inequality-reducing outcome. While higher-income groups experience larger absolute gains, lower-income groups may still derive substantial relative benefits, particularly given their higher vulnerability to catastrophic health expenditures and limited access to healthcare.^15^ From a policy perspective, this indicates that increasing the HPL can generate benefits across the socioeconomic gradient, even if the magnitude of these benefits varies.

The sensitivity analysis reinforces the robustness of these findings, demonstrating that even with an 80% tax passthrough, a 20% HPL would avert 514 000 incident cases and ZAR20.1 billion in healthcare costs, while a 120% passthrough would increase benefits to 659 000 cases and ZAR25.3 billion. These variations underscore the pivotal role of tax passthrough in determining the effectiveness of SSB taxes. Producer pricing strategies, such as undershifting or overshifting taxes, significantly influence consumer behaviour and, consequently, health outcomes.^35^ For instance, overshifting, where prices increase beyond the tax increment, amplifies consumption reductions, as observed in Mexico’s SSB tax implementation.^37^ Policymakers must therefore monitor market responses to ensure that tax increases are fully passed through to retail prices, maximising the reduction in SSB consumption. This is particularly relevant in South Africa, where industry responses to the HPL have included partial passthrough, potentially diluting its impact.^17^ These projections assume that all other factors remain constant and should therefore be interpreted as indicative rather than predictive of real-world outcomes. Future changes in diabetes prevention and treatment, including the potential increased availability of therapies such as glucagon-like peptide-1 receptor agonists, as well as broader shifts in the healthcare landscape, may influence long-term disease trends and healthcare costs.

Fiscally, the ZAR15.1 billion in forgone revenue from 2018/2019 to 2023/2024 represents a missed opportunity to fund public health interventions, such as diabetes prevention programmes, nutritional education, or subsidies for healthier food options. These revenues could bolster South Africa’s strained health system, which faces an annual diabetes-related economic burden of up to ZAR29 billion.^9^ The revenue loss is most pronounced in higher-income quintiles (quintiles 4 and 5), where SSB consumption is higher, suggesting that a 20% HPL could generate significant resources while targeting populations with greater purchasing power. This aligns with global evidence that SSB taxes can serve as dual-purpose instruments, simultaneously reducing consumption and generating revenue for health system strengthening.^18^ For example, the Philippines’ SSB tax has successfully funded universal healthcare initiatives, demonstrating the potential of such policies to address both health and fiscal challenges.^20^

The decline in potential revenue over time, from ZAR8 billion in 2018/2019 to ZAR2.8 billion in 2023/2024, highlights the erosion of the HPL’s real value due to inflation. The current tax rate of ZAR0.0221 per gram of sugar, unchanged since 2019, has reduced the effective tax burden to approximately 8% by June 2025. This underscores the need for regular adjustments to the HPL, such as indexing to inflation, to maintain its effectiveness, as recommended by the WHO.^18^ Failure to adjust the tax rate risks diminishing its impact on consumption and revenue, as observed in other jurisdictions where static tax rates have lost efficacy over time.^38^ Given that health taxes also form part of domestic revenue sources for health, our results show that static tax rate limits revenue generation for health.

South Africa’s experience with the HPL since its introduction in 2018 demonstrates that SSB taxes can reduce consumption, with early evaluations showing a 29% reduction in urban household SSB purchases postimplementation.^17^ However, the current 11% rate is insufficient to achieve optimal health outcomes, particularly given the WHO’s 20% threshold for meaningful consumption reductions.^39^ Increasing the HPL to 20% would align South Africa with global best practices, such as those in Mexico and the UK, where higher SSB taxes have led to significant reductions in consumption and obesity-related outcomes.^40 41^ Moreover, the ECEA approach highlights the policy’s potential to generate benefits across income groups, while also contributing to reductions in disease burden and healthcare costs in more vulnerable populations.

Despite these benefits, implementing a higher HPL may face challenges, including industry pushback and public perceptions of regressivity. The beverage industry’s influence was evident in the HPL’s initial reduction from 20% to 11% following consultations in 2017. To counter such resistance, policymakers could adopt strategies from other countries, such as earmarking tax revenues for public health initiatives to enhance public support.^42^ For example, in the UK, SSB tax revenues fund school-based health programmes, increasing public acceptance.^43^ Additionally, transparent communication framing the HPL as a health promotion measure, rather than a punitive tax, could mitigate concerns about regressivity, particularly if paired with subsidies for healthier alternatives.

### Limitations

Although this study provides robust evidence on the health and fiscal costs of not raising South Africa’s HPL to 20%, using ECEA with locally derived data, distributional equity considerations across income quintiles, a 20-year proportional multistate lifetable model, and comprehensive sensitivity analyses, it has limitations. First, the assumption of constant physical activity levels may not fully capture changes in energy expenditure that could influence BMI and diabetes risk. Emerging evidence suggests that lifestyle interventions, including physical activity, can amplify the effects of dietary changes.^44^ Second, the reliance on price elasticity estimates from Saxena *et al*^20^ may not reflect recent shifts in consumer behaviour or market dynamics, particularly post-COVID-19 economic changes. Third, the focus on type 2 diabetes alone underestimates the broader health benefits of reduced SSB consumption, such as decreased risks of cardiovascular diseases, dental caries and certain cancers.^13 45^ Fourth, the model does not account for industry reformulation of SSBs to reduce sugar content, which could mitigate the tax’s impact on consumption but still improve health outcomes, as observed in the UK’s soft drinks industry levy.^46^ Finally, the analysis does not consider potential substitution effects, where consumers may switch to untaxed high-calorie SSBs, potentially offsetting health benefits.^47^

Future research should prioritise incorporating industry responses, including reformulation and product innovation, into modelling frameworks to better capture the full impact of SSB taxation. In addition, further work is needed to generate updated, nationally representative estimates of behavioural and epidemiological parameters and to examine how these relationships evolve over time. Expanding analyses to include additional NCD outcomes and broader dietary substitution patterns would further enhance the robustness and policy relevance of future studies.

## Conclusions

In conclusion, increasing the HPL to align with the WHO’s recommended 20% threshold has the potential to generate substantial health and economic benefits in South Africa. While these benefits accrue across all income groups, they are not distributed equally, with higher-income populations experiencing larger absolute gains. Nonetheless, the policy remains broadly beneficial, as lower-income groups also experience reductions in disease burden and financial risk. These findings highlight the importance of considering both aggregate and distributional effects when evaluating fiscal health policies. Strengthening the HPL should therefore be viewed as a valuable component of a comprehensive strategy to address the growing burden of diet-related NCDs in South Africa.

## Supplementary material

10.1136/bmjph-2025-004415 online supplemental file 1

## Data Availability

Data are available in a public, open access repository.
