# Supplementary material for: Estimating the cost of not setting the Health Promotion Levy at 20% in South Africa: an extended cost-effectiveness analysis
Source: BMJ Public Health. 2026 Jul 9;4(3):e004415. doi: 10.1136/bmjph-2025-004415 (PMC13358265; doi:10.1136/bmjph-2025-004415)
Supplement: online supplemental file 1 [file bmjph-4-3-s001.docx]

**Supplementary file**

**Table S1: Estimates of the potential effect of not setting the HPL at 20% effective tax rate, 80% tax passthrough**

| **By gender** | | | | | | | | | | | | | | | | | | | | | | | | |
| --- | --- | --- | --- | --- | --- | --- | --- | --- | --- | --- | --- | --- | --- | --- | --- | --- | --- | --- | --- | --- | --- | --- | --- | --- |
|  | | **At 20% effective tax rate** | | | | | | | | **At 11% effective tax rate** | | | | | | | | **Cost of Inaction** | | | | | | |
| **Quintile** | **Gender** | **Incident Cases** | | **Prevalence** | | **Deaths** | | **Heath Costs (ZAR)** | | **Incident Cases** | | **Prevalence** | | **Deaths** | | **Health Costs (ZAR)** | | **Incident Cases** | | **Prevalence** | | **Deaths** | | **Health Costs (ZAR)** |
| 1 | Male | -59 735 | | -23 921 | | -2 588 | | -2 234 770 055 | | -41 188 | | -16 872 | | -1 807 | | -1 573 132 773 | | -18 547 | | -7 050 | | -781 | | -661 637 282 |
|  | Female | -164 525 | | -90 541 | | -6 409 | | -7 117 204 188 | | -109 154 | | -61 009 | | -4 273 | | -4 805 296 147 | | -55 371 | | -29 532 | | -2 135 | | -2 311 908 041 |
| 2 | Male | -65 785 | | -25 963 | | -2 829 | | -2 356 835 483 | | -29 990 | | -12 297 | | -1 355 | | -1 125 300 632 | | -35 795 | | -13 665 | | -1 474 | | -1 231 534 851 |
|  | Female | -146 004 | | -75 257 | | -6 566 | | -6 219 922 912 | | -71 179 | | -38 609 | | -3 334 | | -3 202 406 984 | | -74 825 | | -36 648 | | -3 232 | | -3 017 515 928 |
| 3 | Male | -67 216 | | -26 938 | | -2 868 | | -2 429 435 711 | | -38 540 | | -15 930 | | -1 684 | | -1 443 305 474 | | -28 676 | | -11 009 | | -1 184 | | -986 130 237 |
|  | Female | -135 860 | | -71 796 | | -5 777 | | -5 748 186 102 | | -77 941 | | -42 662 | | -3 393 | | -3 429 383 826 | | -57 919 | | -29 133 | | -2 384 | | -2 318 802 276 |
| 4 | Male | -74 921 | | -30 035 | | -3 179 | | -2 748 721 455 | | -36 864 | | -15 282 | | -1 627 | | -1 406 955 499 | | -38 058 | | -14 752 | | -1 552 | | -1 341 765 955 |
|  | Female | -143 121 | | -77 434 | | -5 799 | | -6 141 840 245 | | -72 847 | | -40 966 | | -3 066 | | -3 275 727 861 | | -70 274 | | -36 467 | | -2 733 | | -2 866 112 385 |
| 5 | Male | -111 442 | | -42 727 | | -4 934 | | -4 199 224 972 | | -61 078 | | -24 247 | | -2 758 | | -2 384 739 544 | | -50 364 | | -18 480 | | -2 176 | | -1 814 485 428 |
|  | Female | -180 819 | | -96 045 | | -7 671 | | -7 962 194 708 | | -96 407 | | -52 712 | | -4 274 | | -4 403 272 392 | | -84 412 | | -43 333 | | -3 397 | | -3 558 922 316 |
| **Overall** |  | **-1 149 429** | | **-560 656** | | **-48 619** | | **-47 158 335 831** | | **-635 189** | | **-320 586** | | **-27 572** | | **-27 049 521 132** | | **-514 241** | | **-240 069** | | **-21 047** | | **-20 108 814 699** |
| **Total (sum of males and females)** | | | | | | | | | | | | | | | | | | | | | | | | |
|  | **At 20% effective tax rate** | | | | | | | | **At 11% effective tax rate** | | | | | | | | **Cost of Inaction** | | | | | | | |
| **Quintile** | **Incident Cases** | | **Prevalence** | | **Deaths** | | **Heath Costs (ZAR)** | | **Incident Cases** | | **Prevalence** | | **Deaths** | | **Health Costs (ZAR)** | | **Incident Cases** | | **Prevalence** | | **Deaths** | | **Health Costs (ZAR)** | |
| 1 | -224 260 | | -114 462 | | -8 996 | | -9 351 974 244 | | -150 342 | | -77 880 | | -6 080 | | -6 378 428 921 | | -73 918 | | -36 581 | | -2 916 | | -2 973 545 323 | |
| 2 | -211 789 | | -101 220 | | -9 395 | | -8 576 758 394 | | -101 169 | | -50 907 | | -4 689 | | -4 327 707 616 | | -110 620 | | -50 313 | | -4 706 | | -4 249 050 778 | |
| 3 | -203 077 | | -98 734 | | -8 645 | | -8 177 621 813 | | -116 482 | | -58 592 | | -5 078 | | -4 872 689 300 | | -86 595 | | -40 142 | | -3 567 | | -3 304 932 513 | |
| 4 | -218 043 | | -107 469 | | -8 978 | | -8 890 561 700 | | -109 711 | | -56 249 | | -4 693 | | -4 682 683 360 | | -108 332 | | -51 220 | | -4 285 | | -4 207 878 340 | |
| 5 | -292 261 | | -138 772 | | -12 605 | | -12 161 419 680 | | -157 485 | | -76 959 | | -7 032 | | -6 788 011 935 | | -134 776 | | -61 813 | | -5 573 | | -5 373 407 744 | |
| **Overall** | **-1 149 429** | | **-560 656** | | **-48 619** | | **-47 158 335 831** | | **-635 189** | | **-320 586** | | **-27 572** | | **-27 049 521 132** | | **-514 241** | | **-240 069** | | **-21 047** | | **-20 108 814 699** | |

**Table S2: Estimates of the potential effect of not setting the HPL at 20% effective tax rate, 120% tax passthrough**

| **By gender** | | | | | | | | | | | | | | | | | | | | | | | | |
| --- | --- | --- | --- | --- | --- | --- | --- | --- | --- | --- | --- | --- | --- | --- | --- | --- | --- | --- | --- | --- | --- | --- | --- | --- |
|  | | | **At 20% effective tax rate** | | | | | | | | **At 11% effective tax rate** | | | | | | | | **Cost of Inaction** | | | | | |
| **Quintile** | **Gender** | | **Incident Cases** | | **Prevalence** | | **Deaths** | | **Heath Costs (ZAR)** | | **Incident Cases** | | **Prevalence** | | **Deaths** | | **Health Costs (ZAR)** | | **Incident Cases** | | **Prevalence** | **Deaths** | **Health Costs (ZAR)** | |
| 1 | Male | | -89 958 | | -35 418 | | -3 873 | | -3 311 679 414 | | -51 258 | | -20 750 | | -2 224 | | -1 936 168 027 | | -38 700 | | -14 668 | -1 649 | -1 375 511 388 | |
|  | Female | | -227 337 | | -123 302 | | -9 014 | | -9 659 065 340 | | -138 036 | | -76 503 | | -5 368 | | -6 020 652 874 | | -89 301 | | -46 798 | -3 646 | -3 638 412 466 | |
| 2 | Male | | -85 776 | | -33 229 | | -3 708 | | -3 025 436 621 | | -46 206 | | -18 608 | | -2 008 | | -1 690 385 466 | | -39 570 | | -14 622 | -1 700 | -1 335 051 156 | |
|  | Female | | -182 023 | | -91 472 | | -8 362 | | -7 609 781 870 | | -106 679 | | -56 463 | | -4 796 | | -4 658 948 248 | | -75 344 | | -35 009 | -3 565 | -2 950 833 622 | |
| 3 | Male | | -80 082 | | -31 630 | | -3 442 | | -2 858 194 442 | | -48 643 | | -19 862 | | -2 095 | | -1 794 537 378 | | -31 439 | | -11 768 | -1 348 | -1 063 657 064 | |
|  | Female | | -163 136 | | -84 917 | | -7 025 | | -6 815 480 221 | | -99 911 | | -54 021 | | -4 248 | | -4 326 310 853 | | -63 225 | | -30 895 | -2 777 | -2 489 169 368 | |
| 4 | Male | | -107 137 | | -42 039 | | -4 573 | | -3 851 235 771 | | -53 172 | | -21 703 | | -2 278 | | -1 989 186 914 | | -53 965 | | -20 336 | -2 295 | -1 862 048 858 | |
|  | Female | | -193 543 | | -101 875 | | -8 077 | | -8 097 504 643 | | -103 694 | | -57 295 | | -4 210 | | -4 555 126 386 | | -89 849 | | -44 580 | -3 867 | -3 542 378 257 | |
| 5 | Male | | -134 189 | | -50 727 | | -5 990 | | -4 994 196 191 | | -66 776 | | -26 179 | | -3 030 | | -2 579 325 643 | | -67 413 | | -24 548 | -2 960 | -2 414 870 548 | |
|  | Female | | -217 900 | | -114 964 | | -9 241 | | -9 512 623 469 | | -107 620 | | -58 810 | | -4 664 | | -4 902 889 770 | | -110 280 | | -56 154 | -4 577 | -4 609 733 698 | |
| **Overall** |  | | **-1 481 082** | | **-709 573** | | **-63 305** | | **-59 735 197 983** | | **-821 996** | | **-410 194** | | **-34 920** | | **-34 453 531 558** | | **-659 086** | | **-299 379** | **-28 384** | **-25 281 666 424** | |
| **Total (sum of males and females)** | | | | | | | | | | | | | | | | | | | | | | | | |
|  | | **At 20% effective tax rate** | | | | | | | | **At 11% effective tax rate** | | | | | | | | **Cost of Inaction** | | | | | | |
| **Quintile** | | **Incident Cases** | | **Prevalence** | | **Deaths** | | **Heath Costs (ZAR)** | | **Incident Cases** | | **Prevalence** | | **Deaths** | | **Health Costs (ZAR)** | | **Incident Cases** | | **Prevalence** | | **Deaths** | | **Health Costs (ZAR)** |
| 1 | | -317 295 | | -158 720 | | -12 887 | | -12 970 744 754 | | -189 295 | | -97 254 | | -7 592 | | -7 956 820 901 | | -128 001 | | -61 466 | | -5 295 | | -5 013 923 854 |
| 2 | | -267 799 | | -124 702 | | -12 069 | | -10 635 218 491 | | -152 885 | | -75 071 | | -6 804 | | -6 349 333 714 | | -114 914 | | -49 631 | | -5 265 | | -4 285 884 778 |
| 3 | | -243 218 | | -116 547 | | -10 467 | | -9 673 674 663 | | -148 554 | | -73 883 | | -6 343 | | -6 120 848 231 | | -94 664 | | -42 664 | | -4 124 | | -3 552 826 432 |
| 4 | | -300 680 | | -143 914 | | -12 650 | | -11 948 740 415 | | -156 867 | | -78 998 | | -6 488 | | -6 544 313 300 | | -143 814 | | -64 916 | | -6 162 | | -5 404 427 115 |
| 5 | | -352 089 | | -165 691 | | -15 231 | | -14 506 819 659 | | -174 396 | | -84 989 | | -7 694 | | -7 482 215 413 | | -177 693 | | -80 702 | | -7 537 | | -7 024 604 246 |
| **Overall** | | **-1 481 082** | | **-709 573** | | **-63 305** | | **-59 735 197 983** | | **-821 996** | | **-410 194** | | **-34 920** | | **-34 453 531 558** | | **-659 086** | | **-299 379** | | **-28 384** | | **-25 281 666 424** |
